# Loss of IGF1R in Human Astrocytes Alters Complex I Activity and Support for Neurons

**DOI:** 10.1016/j.neuroscience.2018.07.029

**Published:** 2018-10-15

**Authors:** Laura E. Ratcliffe, Irina Vázquez Villaseñor, Luke Jennings, Paul R. Heath, Heather Mortiboys, Aurelie Schwartzentruber, Evangelia Karyka, Julie E. Simpson, Paul G. Ince, Claire J. Garwood, Stephen B. Wharton

**Affiliations:** Sheffield Institute for Translational Neuroscience, University of Sheffield, 385A Glossop Road, Sheffield S10 2HQ, UK

**Keywords:** ACM, Astrocyte conditioned media, AD, Alzheimer’s disease, GFAP, Glial fibrillary acidic protein, IGF1, insulin-like growth factor, LUHMES, Lund human mesencephalic cells, NACM, Neuron astrocyte conditioned media, NADH, nicotinamide adenine dinucleotide, astrocytes, IGF1, oxidative stress, metabolism, mitochondria

## Abstract

The insulin/insulin-like growth factor 1 (IGF1) signaling pathways are implicated in longevity and in progression of Alzheimer’s disease. Previously, we showed that insulin-like growth factor 1 receptor (IGF1R) and downstream signaling transcripts are reduced in astrocytes in human brain with progression of Alzheimer’s neuropathology and developed a model of IGF1 signaling impairment in human astrocytes using an IGF1R-specific monoclonal antibody, MAB391. Here, we have established a novel human astrocyte-neuron co-culture system to determine whether loss of astrocytic IGF1R affects their support for neurons. Astrocyte-neuron co-cultures were developed using human primary astrocytes and differentiated Lund Human Mesencephalic Cells (LUHMES). Neurite outgrowth assays, performed to measure astrocytic support for neurons, showed astrocytes provided contact-mediated support for neurite outgrowth. Loss of IGF1R did not affect neurite outgrowth under control conditions but when challenged with hydrogen peroxide IGF1R-impaired astrocytes were less able to protect LUHMES. To determine how loss of IGF1R affects neuronal support MAB391-treated astrocytes were FACS sorted from GFP-LUHMES and their transcriptomic profile was investigated using microarrays. Changes in transcripts involved in astrocyte energy metabolism were identified, particularly NDUFA2 and NDUFB6, which are related to complex I assembly. Loss of complex I activity in MAB391-treated astrocytes validated these findings. In conclusion, reduced IGF1 signaling in astrocytes impairs their support for neurons under conditions of stress and this is associated with defects in the mitochondrial respiratory chain in astrocytes.

## Highlights

• We have established a novel human astrocyte-neuron co-culture system.
• Astrocytes provided contact-mediated support for neurite outgrowth.
• IGF1R-impaired astrocytes are less able to protect neurons under stress conditions.
• Microarray analysis of these astrocytes identified changes in energy metabolism.

## Introduction

The IGF1 signalling pathway involves a complex network of intracellular interactions that are essential for cell survival, growth and metabolism. IGF1 can enter the brain by transcytosis across the blood brain barrier (BBB) (Nishijima et al., 2010) or be produced centrally by neurons and glia (Adamo et al., 1988, Cao et al., 2011, Suh et al., 2013). IGF1 binding to homodimers of IGF1R, or heterodimers comprising IGF1R and insulin receptor (IR), induces a conformational change resulting in autophosphorylation of the intracellular beta kinase domain. This triggers downstream phosphorylation cascades that result in activation of either protein kinase B (Akt) or the extracellular-regulated kinase (Erk) pathway. IGF1 is a more potent activator of the phosphoinositide-3-kinase (PI3K)-Akt pathway, which typically regulates metabolism and growth, and is a weak activator of the Erk pathway, which has a more mitogenic role (Weng et al., 2001, Clerk et al., 2006). In the brain, activation of these divergent signalling pathways by IGF1 is important in promoting neurogenesis, neurite outgrowth and increasing synaptic coverage (Hansson et al., 1986, Cheng et al., 2003).

During aging there is a gradual decline in growth hormone and IGF1 which is associated with a decrease in muscle mass and increase in fat deposition, as well as other age-associated changes (Maggio et al., 2013). Although reduction of IGF1 occurs as a consequence of aging, there is extensive literature showing that reduced signaling through this pathway extends lifespan but paradoxically contributes to progression of age-related diseases, such as cardiovascular and neurodegenerative diseases. Impairment of IGF1 signaling has been demonstrated in Alzheimer’s Disease (AD), particularly in neurons. Altered IGF1 signaling has also been reported in astrocytes in relation to Alzheimer’s type neuropathology (Moloney et al., 2010, Simpson et al., 2011). In rodent astrocytes, IGF1 signaling is important for glucose uptake, regulation of glutamate transport and protection against oxidative stress in the brain (Hamai et al., 1999, Suzuki et al., 2001, Genis et al., 2014). In mice, reduced IGF1 levels in the serum and cerebral cortex causes neurovascular uncoupling which is linked to loss of metabotropic receptors and impaired release of vasodilator mediators, such as arachidonic acid (Toth et al., 2015). These studies suggest that astrocytic IGF1 signaling is important for maintaining the support and protection of neurons.

Previously, we developed an *in vitro* model of impaired IGF1-signaling in human astrocytes to explore how reduced input through this pathway affects function. However no changes in phenotype were observed when these astrocytes were grown in monoculture (Garwood et al., 2015). Astrocytes mature functionally when grown with neurons, as demonstrated by upregulation of astrocyte-specific proteins such as glutamate transporters (Swanson et al., 1997) and connexin 43/30 (Koulakoff et al., 2008) and changes in their transcriptomic profile that are more representative of astrocytes found *in vivo* (Goudriaan et al., 2014). In addition, IGF1 enhances neurite outgrowth only when astrocytes are present in co-culture with neurons, suggesting that the IGF1 signaling pathway in astrocytes provides support for neurons (Ang et al., 1992). Thus a co-culture model is more likely to replicate aspects of the systems biology dysfunction present in AD brain.

We hypothesized that reduced IGF1 signaling in astrocytes impairs their ability to provide support and protection for neurons. We developed a human astrocyte-neuron co-culture system and applied an IGF1R monoclonal antibody to reduce the IGF1-receptor in astrocytes. We show that, although this does not impair neurite outgrowth under basal conditions, it impairs astroglial-mediated neural support under stress conditions. To further define how reduced IGF1R in astrocytes may alter their ability to support neurons, we used microarray gene expression analysis of astrocytes separated from the co-cultures to explore alterations in gene expression pathways.

## Experimental procedures

Unless otherwise stated all materials were obtained from Sigma Aldrich (Poole, Dorset, UK).

### Primary human astrocytes, LUHMES, GFP LUHMES, fibroblasts

Primary human astrocytes were obtained from ScienCell Research Laboratories (Carlsbad, CA, US). Astrocytes were expanded in Human Astrocyte Media (AM) (ScienCell Research Laboratories) supplemented with fetal bovine serum (FBS), penicillin streptomycin and Astrocyte Growth Supplement (all ScienCell Research Laboratories), prior to commencing experimental work astrocytes were cultured in a 50:50 mix of F10:MEMα media (Gibco) supplemented with 10% FBS (Biosera, South America origin) and 1% penicillin streptomycin (Lonza, Walkersville). To reduce IGF1R levels, astrocytes were treated with 11 μg/ml of an IGF1R-specific monoclonal antibody, MAB391 (R&D Systems, MN, USA), for 24 h. This concentration is the known EC50 value determined by R&D systems and was used previously to reduce IGF1R (Garwood et al., 2015). After 24 h, cells were washed with 1 × PBS and cells were cultured for a further 72 h in fresh F10:MEMα media. An IgG isotype control, MAB002 (R&D Systems, MN, USA) was also included.

Lund human mesencephalic cells (LUHMES) are conditionally-immortalized neuronal precursor cells that can be differentiated over a 5-day period into post-mitotic neurons (Scholz et al., 2011). Here they were used as a neuronal readout of astrocyte support. LUHMES were grown on cell culture flasks in proliferation medium consisting of DMEM/F12 GlutaMAX™ supplement medium (Gibco), N2 supplement (Gibco) and 40 ng/ml recombinant basic fibroblast growth factor (FGF) (Peprotech, NJ, USA). When cells were 50–60% confluent they were differentiated by replacing media with differentiation media consisting of DMEM/F12 GlutaMAX™ supplement medium, N2 supplement and 1 μg/ml tetracycline. After two days, LUHMES were trypsinized and replated onto the appropriate plates for a further 3 days before becoming fully differentiated.

GFP LUHMES were generated by transducing non-differentiated LUHMES with GFP-expressing lentiviral particles (LV-GFP). GFP expression was under the control of a PGK promoter. Low passage (p3-p5) proliferating LUHMES, seeded at 1.5x10^6^ cells per T75 flask, were transduced with LV-GFP for 24 h. Media were replaced with fresh proliferation media and left for a further 72 h before carrying out experiments.

Fibroblasts were obtained from NIGMS Human Genetic Cell Repository at the Coriell Institute for Medical Research (line GM09400) and were cultured in Minimum essential medium supplemented with 10% FBS (Labtech International, Heathfield, UK), 1% P/S, 1% MEM non-essential amino acids, 1% MEM vitamins, 1% sodium pyruvate and 60 μg/ml uridine. Fibroblasts were used as a control to ensure that effects on neurite outgrowth were specific to astrocytes.

### Co-cultures

Astrocytes were co-cultured with LUHMES/GFP LUHMES based on the strict differentiation protocol of these cells. Initial neurite outgrowth assays were performed to assess astrocyte support for LUHMES under control conditions. Astrocytes were seeded onto fibronectin (1 μg/ml)-coated 24-well plates (30,000 astrocytes/well) and differentiation media were added to a separate flask of LUHMES. 2 days later LUHMES were replated onto astrocytes (7500 cells/well) and the co-cultures were then grown in differentiation media for 24 h. LUHMES were also cultured in astrocyte conditioned media (ACM) and neuron-astrocyte conditioned media (NACM) to determine whether astrocytes support was contact mediated or was through the release of soluble factors into the media. The fibroblast-LUHMES co-culture system was set up in a similar manner but with fibroblasts seeded onto fibronectin-coated plates at a slightly higher density (50,000 fibroblasts/well).

For co-culture experiments with MAB391-treated astrocytes the protocol was as follows: On day 1 astrocytes were seeded onto fibronectin-coated plates and differentiation media were added to a separate flask of GFP-LUHMES. On day 2, astrocytes were treated with 11 μg/ml MAB391 or MAB002 for 24 hour. On day 3 GFP LUHMES were replated onto treated astrocytes, and at this point the monoclonal antibody was removed. Co-cultures were then maintained in differentiation media for 72 h, after which co-cultures were treated with 50 μM H_2_O_2_ for 2 h.

### Preparation of astrocyte lysates

Following treatments, medium was removed and cells were washed with ice-cold PBS and lysed directly in extra strong lysis buffer (100 mM Tris-HCl (pH 7.5), 0.5% (*w/v*) sodium dodecyl sulfate (SDS), 0.5% (*w/v*) sodium deoxycholate, 1% (*v/v*) Triton X-100, 75 mM sodium chloride (NaCl), 10 mM ethylenediaminetetraacetric acid, 2 mM sodium orthovanadate, 1.25 mM sodium fluoride, protease inhibitor cocktail and PhosStop (both Roche, Basel, Switzerland). Lysates were incubated on ice for 25 min, sonicated and then centrifuged at 17 000 g (_av_) for 30 min at 4 °C. A BCA protein assay kit (ThermoFisher Scientific, Waltham, MA, USA) was used to determine protein concentration.

### SDS-PAGE and immunoblotting

40 μg of protein was loaded onto 8.5% (*w/v*) SDS-PAGE gels and electrophoretically transferred to nitrocellulose membrane (GE Healthcare, little Chalfont, Bucks, UK). Membranes were blocked with Western Ezier Blocking buffer (GenDEPOT, Barker, TX, UK) and then probed with primary antibody, followed by horseradish peroxidase (HRP)-conjugated secondary antibodies (DAKO, Copenhagen, Denmark) and ECL (EZ-ECL Biological Industries Israel). Proteins were detected using the G:Box Chemi-XT CCD Gel imaging system (Syngene, Cambridge, UK). Membranes were incubated with the following primary antibodies: IGF1Rβ (1/200; Rabbit polyclonal sc-713, Santa Cruz BioTechnology [SCBT], Dallas, Texas, US), Akt (1/1000; Rabbit polyclonal 4685, CST), phospho Akt S473 (1/2000; Rabbit monoclonal 4060, CST) and α-tubulin (1/200, rabbit, ab18251, Abcam).

### Immunocytochemistry

Cells were fixed in 4% (*w/v*) paraformaldehyde in PBS for 5 min at 37 °C. Fixed cells were permeabilized (0.3% (*v/v*) Triton X-100 in PBS) and then blocked with 3% (*w/v*) bovine serum albumin before incubation with primary antibodies. Co-cultures were first probed with Beta-III-tubulin (1/1000; Chicken polyclonal, Millipore, 2424655) followed by the appropriate species of Alexa-fluor-conjugated secondary antibody (Life technologies). Co-cultures were then incubated with primary antibodies against GFAP (1/1000, Rabbit polyclonal, Abcam Ab7260), Vimentin (1/250, Mouse monoclonal, Abcam, Ab8978) and ALDH1L1 (1/100, Rabbit polyclonal, Abcam, Ab79727) followed by incubation with the appropriate species of secondary antibody. Nuclei were stained with Hoechst 33342 (5 μg/ml bisbenzimide in PBS). Astrocytes in monoculture were also incubated with the primary antibodies detailed above. Images were captured on a on a LV100ND microscope fitted with a DS Ril Eclipse camera (Nikon, Tokyo, Japan).

### MTT assays

MTT (3-[4,5-dimethylthiazol-2-yl]-2,5 diphenyl tetrazolium bromide) assays were performed to determine an appropriate, sublethal, concentration of H_2_O_2_ to stress the co-cultures_._ MTT (0.5 mg/mL) solution (110 μL) was added to each well of a 12 well plate, and the plates were incubated at 37 °C with 5% CO_2_ for 3 h. Afterward, 1.1 ml of 20% SDS in 50% dimethylformamide was added to each well, and the plates were incubated on a mini orbital shaker SSM1 at 150 rpm (Bibby Scientific, Stone, UK) for 3 h until formazan crystals were fully dissolved. The OD of samples was determined by measuring on a plate reader at 570 nm.

### Neurite outgrowth assays

GFP LUHMES were co-cultured with astrocytes for 72 h as described above, before fixation with 4% (*w/v*) paraformaldehyde. Cells were permeabilized (0.3% (*v/v*) Triton X-100 in PBS) and blocked with 3% (*w/v*) bovine serum albumin. Cell nuclei were stained with Hoechst 33342 (5 μg/ml bisbenzimide in PBS) and mounted on slides with fluorescent mounting medium (DakoCytomation). Five images were captured of each coverslip in a clockwise manner (3 coverslips per experimental condition) on a LV100ND microscope fitted with a DS Ril Eclipse camera (Nikon). Neurite length was measured using an ImageJ plugin called Simple Neurite Tracer. To assess astrocytic protection against external stress, 72 h co-cultures were treated with 50 μM of H_2_O_2_ (sublethal concentration, data not shown) for 2 h before fixing cells and assessing neurite lengths.

### Facs

To separate MAB391/IgG-treated astrocytes co-cultured with GFP LUHMES cells were FACS sorted and separated using the FACS ARIA cell sorter (BD Biosciences). Cells were sorted for fluorescence and forward scatter (FSC)/side scatter (SSC) size dimensions. Cells with low fluorescence or cells with overlapping SSC profiles were discarded in order to achieve enriched populations of astrocytes and GFP LUHMES. Cells were collected in Advanced MEM media, centrifuged for 4 min at 400 g (_av)_ and then resuspended in TRIzol® (Life Technologies). PCR analysis was then performed to assess enrichment of cell populations. RNA from FACS sorted cells was isolated using Clean and Concentrator columns (Zymo, Irvine, CA, US). Total RNA was then incubated at 65 °C for 5 min and reverse transcribed at 42 °C for 50 min in a reaction mix containing qScript™ cDNA SuperMix (Quanta, Biosciences, Beverly, US). Amounts of NFL, NeuN, GFAP and β-actin were detected using primers listed in Table 1. PCR was performed in a G-storm Thermal Cycler (G-storm, Somerset, UK) using the following protocol: 94 °C for 30 s, 67 °C for 1 min, 72 °C for 30 s for 35 cycles. Samples were run on a 3% agarose gel containing ethidium bromide at a final concentration of 100 ng/ml. Gels were imaged using the GENI UV light imaging system (Syngene).

**Table 1:** PCR primer details

Primer type	Forward sequence	Reverse Sequence
β-actin	TCCCCCAACTTGAGATGTATGAAG	AACTGGTCTCAAGTCAGTGTACAGG
GFAP	GCAGAAGCTCCAGGATGAAAC	TCCACATGGACCTGCTGTC
NFL	GGCTCTCAGTGTATTGGCTTCTGT	AACCCAGGTCTAGTAAGCAGAAAT
NeuN	ACGATCGTAGAGGACGGAA	AATTCAGGCCCGTAGACTGC

### Microarray analysis

The GeneChip® WT PLUS reagent kit (Affymetrix) was used to produce amplified, fragmented and labeled sense-strand DNA targets from total RNA. In brief RNA was reverse transcribed to produce single-stranded cDNA, double-stranded cDNA was then synthesized to produce a template for *in vitro* transcription, which generated complementary RNA (cRNA). cRNA was used to produce sense strand cDNA (ss-cDNA), which was purified and treated with RNase to hydrolyze the cRNA template. ss-cDNA was fragmented and labeled with biotin before hybridizing to GeneChip® Human Gene ST (GST) Arrays using the GeneChip® Hybridisation, Wash and Stain Kit. All samples were hybridized to arrays on the same day. Samples were then injected and hybridized onto GeneChip® Human GST cartridges and incubated in a GeneChip® hybridization oven 645 at 16 rpm for 16 h at 45 °C. Hybridization, washing and staining was performed using the Fluidics Station 400 and the Gene Chip Operating System (GCOS). The GC3000 7G scanner was used to scan gene chips and the expression console software (Affymetrix) was used to assess the quality of the data. Further analysis was carried out using Qlucore Omics Explorer (Qlucore, Lund, Sweden).

Data were normalized using the Robust Multi-array Average (RMA) and a principal component analysis was performed to determine the intensity distribution and eliminate sample outliers.

## Microarray validation

### PrimeTime® qPCR Assays

Changes in transcript levels identified in the microarray analysis were validated on FACS sorted control and MAB391-treated astrocytes using mini qPCR assays (Integrated DNA Technologies®), listed in Table 2. Each assay mix contained 60 ng of cDNA, 500 nM forward and reverse primer, and a 250 nM probe resuspended in TE buffer (10 mM Tris-HCl, 1 mM EDTA, pH 7.5), 2 × Brilliant III qPCR Master mix and nuclease free dH_2_O. Samples were run on a 2-step profile on a Stratagene MX3000P™ Real Time Thermal Cycler (Agilent Technologies Ltd) and incubated as follows: 10 minutes at 95 °C then 40 cycles of 30 s at 95 °C, 60 s at 60 °C and 60 s at 72 °C. GAPDH was amplified on each plate to normalize expression levels of target genes between different samples using the ΔΔCt calculation (ABI) and to assess assay reproducibility.

**Table 2:** qPCR primer/probe sequence

Gene	PrimeTime ® Assay ID	Ref Seq	Region	Primer Sequence
NDUFA2	Hs.PT.58.38915668	NR_033697	Exon 2-3	5′-AGCACTGAAGTTGTTCAAAGG-3′ 5′-GACTTCATTGAGAAACGCTACG-3′
NDUFB6	Hs.PT.58.20921068.g	NM_002493	Exon1-1	5′-CATCGCCTTCTCAGCTCTC-3′ 5′-CTAGTCCGTAGTTCGAGGGT-3′
TP11	Hs.PT.58.40028166.g	NM_001159287	Exon 3-4	5′-TCCGCAGTCTTTGATCATGC-3′ 5′-CTACTGCCTATATCGACTTCGC-3′
ENO2	Hs.PT.58.578449	NM_001975	Exon7-8	5′-TTCCTTCACCAGCTCCAAG-3′ 5′-CAGAGGTCTACCATACACTCAAG-3′
GAPDH	Hs.PT.39a.22214836	NM_002046	Exon 2-3	5′-TGTAGTTGAGGTCAATGAAGGG-3′ 5′-ACATCGCTCAGACACCATG-3′

### Functional assays

Nicotinamide adenine dinucleotide (NADH)-dependent complex I activity was measured using the complex I enzyme activity microplate assay kit (Abcam, ab109721, Cambridge, UK) and performed according to manufacturer’s protocol. The assay was performed on both mono-cultured astrocytes and co-cultures grown on fibronectin-coated 10-cm dishes and 250 μg of protein was needed per well of the microplate. Extracellular lactate in the co-culture media was measured using the lactate assay kit (Abnova, KA0833, Taiwan) and performed according to the manufacturer’s protocol.

### Statistical analysis

Data were analyzed using either student’s unpaired t-test or a one-way analysis of variance with Tukey’s post-hoc analysis. Prior to performing t-tests data were checked for normality using the D’Agostino and Pearson omnibus normality test (Graphpad Prism 5.0 Software, Graphpad Software Inc., La Jolla, CA, USA).

The Integrated Molecular Pathways Level Analysis (IMPaLA) enrichment tool was used to generate enriched biological pathways from the official gene symbols imported from the microarray.

## Results

### Development of a human astrocyte-neuron co-culture system

**Fig. 1:**
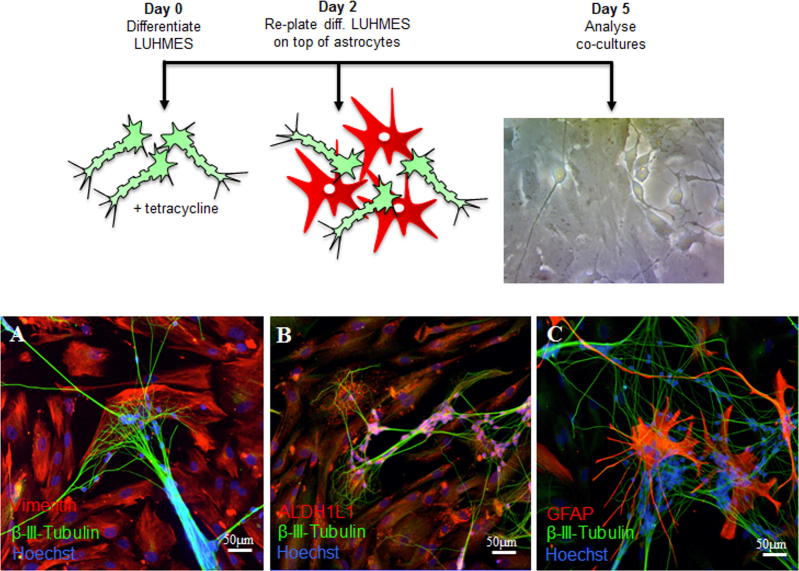
Co-cultured human ScienCell® astrocytes and LUHMES. Schematic depicts the time line for setting up the co-culture system. On day 0 LUHMES were differentiated by addition of tetracycline to the culture medium. Two days later differentiating LUHMES were plated onto control astrocytes. LUHMES and astrocytes were then co-cultured for 72 h prior to fixation, phase contrast image of co-cultured cells is shown. (A-C). Immunocytochemistry of co-cultured LUHMES and astrocytes after 72 h of co-culture. LUHMES were immunolabeled with β-III-tubulin (green), nuclei with Hoechst (blue) and astrocytes were labeled with (A) vimentin, (B) ALDH1L1 and (C) GFAP (all in red). (For interpretation of the references to colour in this figure legend, the reader is referred to the web version of this article.)

In order to assess whether reduced IGF1R levels in astrocytes affected their function a human astrocyte-neuron co-culture system was developed, with LUHMES used as a neuronal readout of astrocyte support. LUHMES develop extensive neurites, form synapses and display spontaneous electrical activity (Scholz et al., 2011). They also express and spontaneously process the amyloid precursor protein and so have been used as a model system for AD (Scholz et al., 2013). LUHMES were differentiated for two days before replating on top of a confluent bed of primary human astrocytes (Fig. 1 schematic). After 72 h in co-culture, astrocytes expressed a range of astrocytic markers including vimentin, aldehyde dehydrogenase 1 family member L1 (ALDH1L1) and glial fibrillary acidic protein (GFAP) (Fig. 1 A-C) while neurons, immunolabeled with β-III-tubulin, extended processes that appeared to localize on top of astrocytes (Fig. 1 A,B). Co-cultured astrocytes displayed a range of morphologies including flat polygonal cells as well as stellate, process-bearing cells.

### Astrocytic support for neurons is contact-dependent

**Fig. 2:**
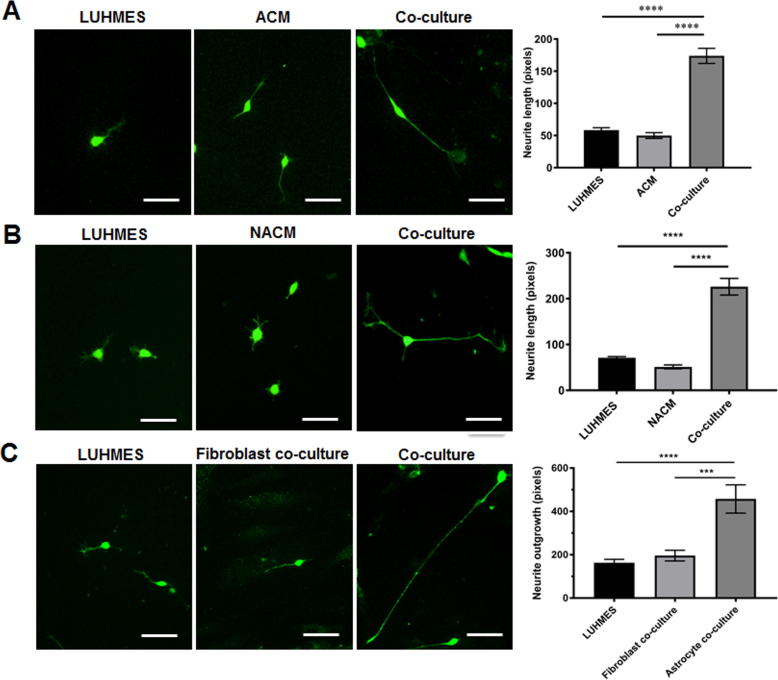
Neurite outgrowth is enhanced by direct contact with astrocytes. LUHMES were cultured with astrocytes, in astrocyte conditioned media (ACM) or in neuron-astrocyte conditioned media (NACM) to determine if enhanced neurite growth was contact-mediated or due to a soluble factor released by astrocytes. (A) Representative images of GFP LUHMES grown in monoculture, in astrocyte-conditioned media (ACM) or in co-culture for 24 h. (B) Representative images of GFP LUHMES grown in monoculture, in neuron-astrocyte conditioned media (NACM) or in co-culture for 24 h. (C) GFP LUHMES were also co-cultured with fibroblasts to confirm enhanced neurite outgrowth was an astrocyte-specific effect Bar charts shows quantification of neurite lengths, data are mean + SEM (*n* = 3, 3 replicates/experiment, One-way ANOVA with post-hoc analysis, ^***^p < 0.001, ^****^ = *p* < 0.0001).

Neurite outgrowth is an important morphological phenotype of neurons and correlates well with neuronal health (Radio and Mundy, 2008, Harrill et al., 2013) We show using this novel co-culture system that astrocytes support neuronal health as demonstrated by a significant increase in neurite length in the presence of astrocytes (Fig. 2A, B, *p* < 0.0001). To determine whether this was mediated by soluble factors released by the astrocytes, or due to specific contacts made in co-culture, neurite length was measured in LUHMES cultured in astrocyte conditioned media (ACM), and in co-cultures. To improve the detection of neurites on a background of astrocytes, LUHMES were transduced with LV-GFP. After 24 h there was a significant increase in neurite outgrowth in co-cultured GFP LUHMES (Fig. 2A, *p* < 0.0001) but not in LUHMES cultured in ACM. To account for the fact that soluble factors released by astrocytes may change when co-cultured with neurons (LUHMES), media was collected from a 24-h co-culture (neuron-astrocyte conditioned media, NACM) and incubated on GFP LUHMES for 24 h prior to measurement of neurite length. Again a significant increase in neurite length was observed only when GFP LUHMES were co-cultured with astrocytes (Fig. 2B, *p* < 0.0001) verifying that the enhancement in neurite outgrowth is contact-mediated. This effect on neurite outgrowth is specific to astrocytes since GFP-LUHMES co-cultured with fibroblasts show no increase in neurite outgrowth (Fig. 2C).

### Loss of IGF1R in astrocytes impairs neuronal support under conditions of stress.

**Fig. 3:**
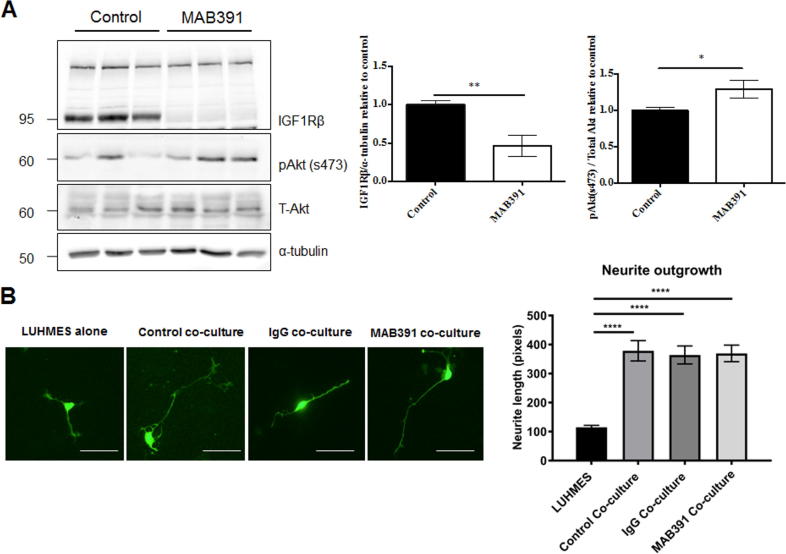
Loss of astrocytic IGF1R does not impair support for neurite outgrowth. (A) Western blots of cell lysates from primary astrocytes. Astrocytes were treated for 24 h with MAB391 to induce IGF1R loss and following a media exchange cultured for a further 72 h. Representative western blots probed with antibodies against IGF1Rβ and pAkt (s473) are shown. Bar charts show quantification of IGF1Rβ relative to α-tubulin and pAkt (s473) relative to total Akt. Data are mean + SEM (*n* = 3, 3 replicates/experiment, unpaired student’s *t*-test ^*^ = *p* < 0.05 and ^**^ = *p* < 0.01). (B) GFP LUHMES were cultured alone or with control, IgG- or MAB391-treated astrocytes for 72 h and neurite outgrowth was assessed (scale bar represents 50 μm). Representative images of GFP LUHMES are shown, bar chart shows quantification of neurite lengths (measured in pixels). Data are mean + SEM (*n* = 3, 3 replicates/experiment, One-way ANOVA with post-hoc analysis, ^****^ = *p* < 0.0001).

An IGF1R-specific monoclonal antibody, MAB391, was used to reduce IGF1R levels in human ScienCell astrocytes (Garwood et al., 2015). MAB391 reduces IGF1R receptor levels by binding to and inducing degradation of the receptor (Hailey et al., 2002, Garwood et al., 2015). Astrocytes were grown in monoculture, treated with MAB391 for 24 h, washed and then cultured for a further 72 h. Immunoblots showed that treatment with MAB391 caused a significant reduction in IGF1R levels (Fig. 3A, *p* < 0.01). There was a significant increase in pAkt (s473) at this timepoint (96 h after MAB391 treatment) (Fig. 3A, *p* < 0.05).

Using neurite outgrowth as a measure of astrocyte support, we investigated the effect of reduced astrocytic IGF1R levels in co-culture. Astrocytes were pre-treated with MAB391 for 24 h, washed and incubated with GFP LUHMES for 72 h before neurite length was measured. Astrocytes significantly enhanced neurite outgrowth after 72 h in co-culture (Fig. 3B, *p* < 0.0001), and this was not affected by loss of IGF1R.

**Fig. 4:**
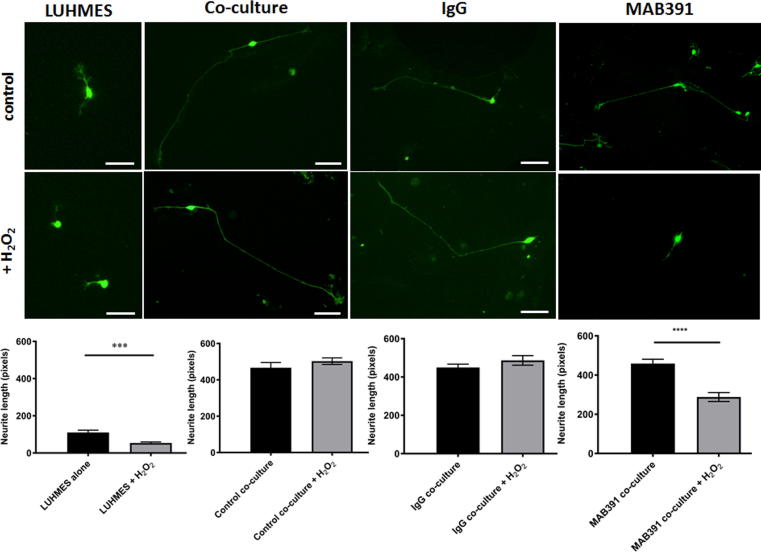
IGF1R impaired astrocytes are less neuroprotective. GFP LUHMES were cultured alone or with control, IgG- or MAB391-treated astrocytes for 72 h. After 72 h co-cultures were treated with 50 μM H_2_O_2_ for 2 h. Representative images of GFP LUHMES are shown, scale bar represents 50 μm. Bar charts show quantification of neurite lengths from these images (measured in pixels). Data are mean + SEM (*n* = 3, 3 replicates/experiment, unpaired student’s *t*-test, ^***^ = *p* < 0.001 and ^****^ = *p* < 0.0001).

Astrocytes can protect neurons from oxidative stress, and experiments using rodent astrocytes suggest that IGF1 signaling plays an important role in this process (Genis et al., 2014). Since oxidative stress increases in the aging brain, and is associated with various neurodegenerative diseases, we assessed whether astrocytes could support neurons under conditions of oxidative stress, and whether loss of IGF1R abrogated the protection offered to neurons. GFP LUHMES in monoculture showed a significant reduction in neurite length after treatment with 50 μM hydrogen peroxide (H_2_O_2_) for 2 h (Fig. 4. *p* < 0.001). This reduction in neurite length was prevented when GFP LUHMES were co-cultured with control astrocytes. However, when GFP LUHMES were co-cultured with MAB391-treated astrocytes this protection was lost, and a significant reduction in neurite length was observed following H_2_O_2_ treatment (Fig. 4, *p* < 0.0001). Astrocytes were also pre-treated with an IgG isotype control, which does not reduce IGF1R levels (Garwood et al., 2015). Treating astrocytes with the IgG isotype control did not affect support for neurite outgrowth under control or stress conditions, suggesting that it is specifically the loss of IGF1R that leads to impaired astrocyte function.

### Loss of IGF1R affects astrocyte energy metabolism

**Fig. 5:**
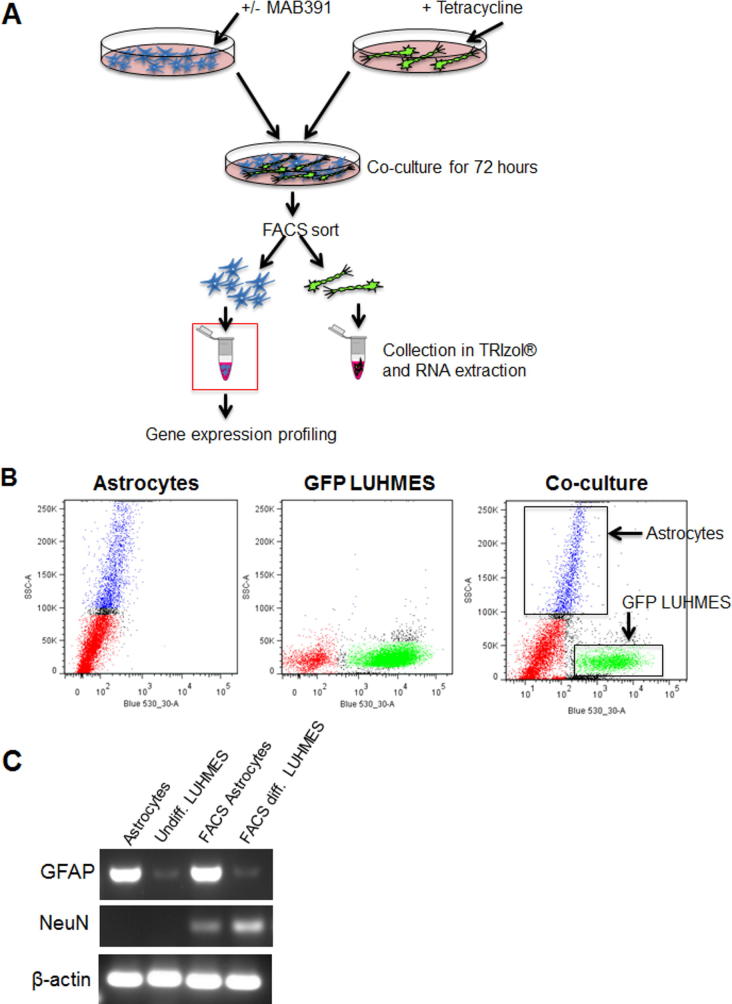
FACS sorting of astrocytes from GFP LUHMES. (A) Astrocytes were treated with or without MAB391 for 24 h, washed and co-cultured with GFP LUHMES for 72 h. Astrocytes and LUHMES were FACS sorted and individually collected in Trizol for RNA extraction. RNA isolated from astrocytes was then subjected to Microarray gene expression analysis. (B) FACS was carried out on astrocytes, GFP LUHMES and co-cultures. Cells were gated according to SSC (side scatter) profile and fluorescence (Blue 530-30-A). (C) DNA gel shows enrichment of astrocytes following FACS sorting of co-cultures; GFAP was used as an astrocytic marker, NeuN as a neuronal marker and β-actin as a loading control. The DNA gel was loaded as follows: astrocyte monoculture, undifferentiated LUHMES monoculture, FACS-sorted astrocytes and FACS-sorted differentiated LUHMES.

Microarray gene expression analysis was performed on FACS sorted MAB391-treated astrocytes separated from co-cultures to assess the underlying transcriptomic changes that may contribute to reduced neuronal support (Fig. 5A, B). A DNA gel was used to assess the separation of astrocytes and LUHMES by checking the expression of astrocyte and neuron-specific transcripts. GFAP expression was elevated in the FACS-sorted astrocytes with minimal expression in FACS-sorted LUHMES, indicating little contamination of the neuronal population with astrocytes. Low levels of NeuN in FACS-sorted astrocytes indicated that astrocytes had been enriched from co-culture during the FACS sorting process (Fig. 5C).

The transcriptomic profile of MAB391-treated astrocytes was determined using Affymetrix GeneChip® Whole Transcript Arrays. The arrays identified 2815 differentially expressed genes in MAB391-treated astrocytes compared to untreated astrocytes, with 1520 transcripts downregulated and 1295 transcripts upregulated (at fold change [FC] = 1.2 and *p* < 0.05). To categorize the biological pathway alterations following MAB391 treatment, pathway enrichment analysis was performed in IMPALA (Table 3, Table 4). Overall, the analysis suggested impaired astrocyte energy metabolism, and more specifically dysregulation of the mitochondrial electron transport chain and glycolysis. Table 5, Table 6 provide lists of genes related to mitochondrial function and glycolysis that were altered in MAB391-treated astrocytes.

**Table 3:** IMPALA Enrichment Pathway analysis for 1520 downregulated transcripts in MAB391-treated astrocytes compared to untreated astrocytes with FC ≥ 1.2 and *p* < 0.05

Pathway	Pathway source	Number of DE genes in the pathway	*p* value
Parkinson’s Disease	KEGG	12	0.0000335
Ubiquitin mediated proteolysis	KEGG	11	0.0000864
TCA cycle and respiratory electron transport	Reactome	11	0.0002250
Electron transport chain	Wikipathways	9	0.0002670
Respiratory electron transport-ATP synthesis by chemiosmotic coupling	Reactome	9	0.0003550
Respiratory electron transport	Reactome	8	0.0003820
Oxidative phosphorylation	KEGG	8	0.0059300
Huntington's Disease	KEGG	10	0.0059700
Mitochondrial electron transport chain	SMPDB	3	0.0065600
Antigen processing	Reactome	9	0.0065900

**Table 4:** IMPALA Enrichment Pathway analysis for 1295 upregulated transcripts in MAB391-treated astrocytes compared to untreated astrocytes with FC ≥ 1.2 and *p* < 0.05

Pathway	Pathway source	Number of DE genes in the pathway	*p* value
Small Ligand GPCRs	Wikipathways	5	0.0000268
Glycolysis	HumanCyc	5	0.0003840
Gluconeogenesis	HumanCyc	5	0.0004650
Glycolysis	Reactome	5	0.0006660
Gluconeogenesis	Reactome	5	0.0012600
Lysosphingolipid and LPA receptors	Reactome	3	0.0012700
Parkin-ubiquitin proteasomal system pathway	Wikipathways	7	0.0024900
Glycine, serine and threonine metabolism	KEGG	5	0.0031000
Rapoport-Luebering glycolytic shunt	HumanCyc	2	0.0038500
Catecholamine biosynthesis	HumanCyc	2	0.0038500

**Table 5:** Transcripts involved in mitochondrial function that are dysregulated in IGF1R impaired astrocytes. Fold change (FC) and *p*-values are also shown

Gene Symbol	Gene Name	FC	*p*-Value
ATP5C1	ATP synthase, H + transporting, mitochondrial F1 complex, gamma polypeptide 1	0.72	5.0E-02
NDUFA1	NADH dehydrogenase (ubiquinone) 1 alpha subcomplex, 1	0.65	3.0E-02
NDUFA2	NADH dehydrogenase (ubiquinone) 1 alpha subcomplex, 2	0.71	4.0E-03
NDUFB3	NADH dehydrogenase (ubiquinone) 1 alpha subcomplex, 3	0.73	3.0E-02
NDUFB6	NADH dehydrogenase (ubiquinone) 1 alpha subcomplex, 6	0.53	3.0E-03
NDUFAB1	NADH dehydrogenase (ubiquinone) 1 alpha/beta subcomplex, 1	0.77	1.0E-03
YME1L1	YME1-like 1	0.58	1.0E-03
ARG2	Arginase, type II	0.76	4.0E-03
ETFA	Electron-transfer-flavoprotein, alpha polypeptide	0.71	4.0E-03
GOLPH3	Golgi phosphoprotein 3	0.82	3.0E-02
MMADHC	Methylmalonic aciduria	0.71	3.0E-04
MGST1	Microsomal glutathione S-transferase 1	0.77	2.0E-02
MRPL3	Mitochondrial ribosomal protein L3	0.76	2.0E-02
MRPL32	Mitochondrial ribosomal protein L32	0.80	2.0E-02
MRPL46	Mitochondrial ribosomal protein L46	0.64	3.0E-02
MRPS18A	Mitochondrial ribosomal protein S18A	0.82	3.0E-02
NLN	Neurolysin	0.69	2.0E-02
NMNAT3	Nicotinamide nucleotide adenylyltransferase 3	0.77	3.0E-02
OGDHL	Oxoglutarate dehydrogenase-like	0.82	2.0E-02
PMPCB	Peptidase (mitochondrial processing) beta	0.75	4.0E-02
PRDX3	Peroxiredoxin 3	0.72	2.0E-03
PPP2R2B	Protein phosphatase 2 (formerly 2A) regulatory subunit B, beta isoform	0.61	2.0E-03
PDK3	Pyruvate dehydrogenase kinase, isozyme 3	0.58	5.0E-02
ROMO1	Reactive oxygen species modulator 1	0.61	1.0E-04
SFXN4	Sideroflexin 4	0.80	2.0E-02
UQCRB	Ubiquinol-cytochrome C reductase binding protein	0.66	6.0E-03
SSBP1	Single-stranded DNA binding protein 1	0.68	5.0E-02
SLC25A14	Solute carrier 25 (mitochondrial carrier, brain)	0.79	2.0E-02
SDHB	Succinate dehydrogenase complex, subunit B	0.76	1.0E-02
SUCLA2	Succinate-coA ligase, ADP forming, beta subunit	0.76	4.0E-02
TXNDC12	Thioredoxin domain containing 12	0.77	6.0E-03
TIMM9	Translocase of inner mitochondrial membrane 9	0.76	3.0E-02
TOMM6	Translocase of outer mitochondrial membrane 6	0.69	1.0E-02

**Table 6:** Transcripts involved in glycolysis that are dysregulated in IGF1R impaired astrocytes. Fold change (FC) and *p*-values are also shown

Gene symbol	Gene Name	FC	*p*-Value
ENO1	Enolase 1	1.23	0.0400
ENO1-AS	Enolase 1-alpha	1.85	0.0030
ENO2	Enolase 2	1.46	0.0100
PDK3	Pyruvate dehydrogenase kinase 3	0.58	0.0500
PGAM2	Phosphoglycerate mutase 2	1.42	0.0006
SLC16A3	Solute Carrier Family 16, Member 3	2.04	0.0200
TPI1	Triosephosphate isomerase 1	1.59	0.0300

**Fig. 6:**
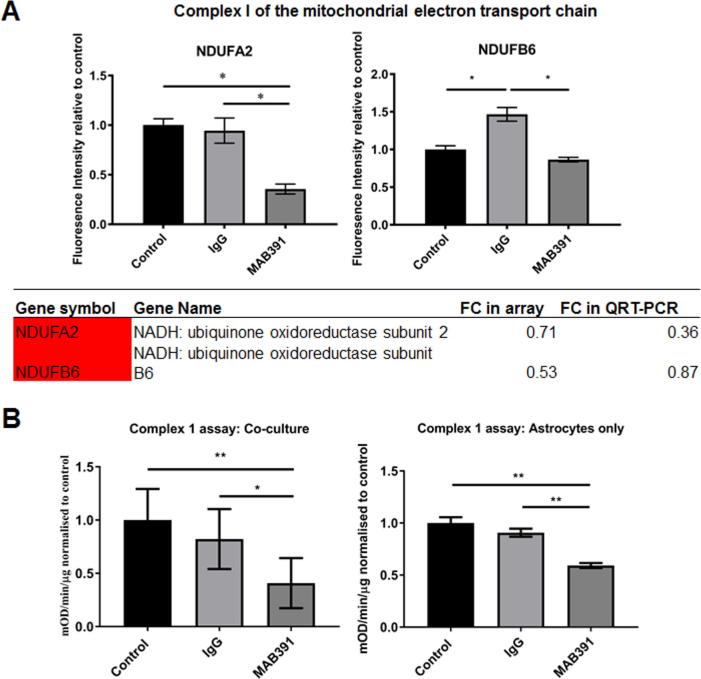
IGF1R impaired astrocytes have defects in complex I. (A) qRT-PCR demonstrated a reduction in *NDUFA2* and *NDUFB6* following MAB391 treatment and table shows fold change in *NDUFA2* and *NDUFB6* in both the microarray analysis and in the qRT-PCR. (B) A complex I assay also showed that MAB391-treated astrocytes were less able to reduce NADH compared to IgG-treated and -untreated astrocytes in co-culture. Data are mean + SEM (*n* = 4, 3 replicates/experiment, One-way ANOVA with post-hoc analysis, ^*^ = *p* < 0.05 and ^**^ = *p* < 0.01). (C) A complex 1 assay was also performed on MAB391-treated mono-cultured human astrocytes to confirm the reduction in co-culture was due to changes in complex-1 activity in astrocytes. Data are mean + SEM and are normalized to control (3 technical repeats, One-way ANOVA with post-hoc analysis, ^**^ = *p* < 0.01).

Since astrocytes can compensate for impaired mitochondrial respiration by switching to glycolysis (Almeida et al., 2001, Bolanos et al., 2008) we assessed whether there was a switch in energy metabolism in IGF1-impaired astrocytes by performing qRT-PCR and functional analysis. Expression of two of the most differentially expressed genes in the electron transport chain, NADH ubiquinone oxidoreductase subunit 2 (*NDUFA2, p* value = 0.003 and FC = 0.71) and B6 (*NDUFB6, p* value = 0.004 and FC = 0.53); and in the glycolysis pathway, triosephosphate isomerase (*TPI1*, *p* value = 0.03 and FC = 1.59) and enolase 2 (*ENO2*, *p* value = 0.01 and FC = 1.46) were assessed in independent FACS-sorted untreated, IgG-treated and MAB391-treated astrocytes. The electron-transport-related transcript, NDUFA2 was downregulated with MAB391 treatment, which is consistent with the array data (Fig. 6A). NDUFB6 showed no significant reduction compared to control, however treatment with IgG alone cause a significant increase which might therefore mask any effect of IGF1R loss on NDUFB6 (Fig. 6A). Changes in the glycolytic transcripts were not clear since TPI1 was significantly downregulated (opposite to the array) and there was an increase in ENO2 transcript levels in MAB391-treated astrocytes, which may be an IgG-specific effect (data not shown).

To assess this putative metabolic switch functionally, a complex I assay and a lactate assay were performed. Complex I activity was assessed since the expression of five genes involved in complex I assembly (NDUFA1, NDUFA2, NDUFB3, NDUFB6 and NDUFAB1) were downregulated following MAB391 treatment. Measurements of complex I activity were initially performed in co-culture, since FACS sorting of astrocytes did not generate sufficient protein to be used in the assay. The activity of complex I was significantly reduced following MAB391 treatment (Fig. 6B) compared to IgG isotype-treated control (*p* < 0.05) and untreated co-cultures (*p* < 0.01). Complex 1 activity was also assessed in mono-cultured astrocytes to confirm that the reduction in complex 1 activity could specifically be attributed to astrocytes, a significant reduction in complex 1 activity was observed in MAB391-treated astrocytes compared to both untreated control (*p* < 0.01) and IgG-treated control (*p* < 0.01) (Fig. 6C). Extracellular lactate levels did not change following IGF1R loss (data not shown). These results indicate that loss of IGF1R in human astrocytes in co-culture induces a defect in the first step in the mitochondrial electron transport chain.

## Discussion

Astrocytes play an important role in maintaining brain health by supporting and protecting neuronal function. Reduced insulin/IGF1 signaling has been demonstrated in neurons in AD and more recently this has been shown in astrocytes (Simpson et al., 2011). Here we show that IGF1R-impaired astrocytes are less able to support and preserve neurite outgrowth following oxidative stress using a novel human astrocyte neuron co-culture system. Microarray analysis indicated that this defect is associated with altered mitochondrial function, particularly impairments in complex I activity.

To assess the impact of reduced IGF1 signaling in human astrocytes we developed a novel co-culture system using human primary astrocytes and conditionally immortalized LUHMES which can be differentiated into post-mitotic neurons (Scholz et al., 2011). Here we show that human primary astrocytes enhance neurite outgrowth, and that this enhancement is contact dependent and is specific to astrocytes.

Treatment with the monoclonal antibody MAB391 resulted in persistent loss of IGF1R, although this is not reflected downstream through Akt. In our previous paper we reported a decrease in pAkt S473 24 h after treatment (Garwood et al., 2015), but here we see an increase 96 h after IGF1R reduction. This likely reflects overcompensation through other receptors which activate Akt either directly or indirectly, these include G-protein-coupled receptors, interleukin receptors, interferon receptors and growth hormone receptors (Murga et al., 1998, Wymann et al., 2003, New et al., 2007). It also highlights that cell signaling pathways and their interactions are not linear and are highly integrated networks.

Loss of IGF1R did not affect the ability of astrocytes to support neurite outgrowth under normal conditions. However, when co-cultures were exposed to H_2_O_2_, there was a significant reduction in neurite length. This is consistent with experiments performed in rodent astrocytes, where IGF1 signaling is important in protecting against reactive oxygen species (ROS) formation and in maintaining support to neurons during oxidative stress (Genis et al., 2014). To explore the underlying mechanisms responsible for this, the transcriptome of MAB391-treated astrocytes isolated from co-culture was investigated using microarray gene expression analysis. This identified a number of biological pathways that may be altered in MAB391-treated astrocytes, including defects in pathways related to astrocyte energy metabolism, and specifically alterations in complex 1 of the mitochondrial respiratory chain.

The five complex I-related transcripts reduced in the array are all found in the “P” module of complex I, which is responsible for anchoring the multi-subunit complex and pumping protons across the inner mitochondrial membrane. Two of these complex I transcripts (*NDUFA2* and *NDUFB6*) were subsequently validated by qPCR. Loss of these specific transcripts may prevent proper assembly of complex I (Yadava et al., 2002, Hoefs et al., 2008, Mimaki et al., 2012) and thus limit its activity. This is supported by the functional assays performed here which showed a loss of NAD^+^-dependent complex I activity in astrocytes. Part of the defect in complex I may be attributed to treatment with the monoclonal antibody as there was a significant reduction in complex I activity following IgG treatment, however there was a further significant reduction after MAB391 treatment. IgG is one of the components in the blood that extravasates from vessels with impairment of the blood brain barrier during neurodegeneration (Paul et al., 2013) and so this result may highlight another mechanism underpinning altered mitochondrial respiration in astrocytes during neurodegeneration. In addition to changes in complex I transcripts, 33 other genes involved in mitochondrial stability and function were downregulated following MAB391 treatment. These findings suggest an overall defect in mitochondrial function in these astrocytes which are not restricted to loss of complex I activity and suggests that a loss of IGF1R results in reduced mitochondrial respiration. This differs from the IR which also signals through pAkt but has actions distinct from IGF1 (Werner and LeRoith, 2014). Loss of this receptor is reported to result in an increase in mitochondrial respiration however, the loss of IR also resulted in reduced glucose uptake and glycolytic rate (Garcia-Caceres et al., 2016) and the increase in mitochondrial respiration might therefore be a compensatory mechanism.

Astrocytes generate most of their ATP through mitochondrial respiration. When this process is inhibited astrocytes upregulate glycolysis to maintain levels of ATP and supply lactate to neurons as an energy substrate for oxidative phosphorylation (Pellerin and Magistretti, 1994, Marcillac et al., 2011, Brix et al., 2012, Kasparov, 2016, Machler et al., 2016). However there is also evidence to suggest that neuronal oxidative phosphorylation is not dependent on lactate supplied by astrocytes (Hall et al., 2012). Although changes in expression of the glycolytic genes *TPI1* and *ENO2* were identified in the array these did not validate in our experiments and there was no obvious effect on extracellular levels of lactate following MAB391 treatment.

Several mechanisms may contribute to complex I dysregulation following IGF1R loss. Loss of IGF1R has been associated with increased GLUT1 expression and glucose uptake in rodent astrocytes (Hernandez-Garzon et al., 2016). If this process is not properly regulated more glycolytically produced NADH will ‘overdrive’ complex I, resulting in increased leakage of electrons and superoxide formation (Pryde and Hirst, 2011). Increased ROS generation can cause oxidative damage to mitochondrial proteins and DNA, including those related to complex I. Loss of astrocyte complex I activity may be the underlying mechanism responsible for reduced protection of neurons against H_2_O_2_. Inhibition of mitochondrial activity with fluorocitrate, causes reduced ATP production and changes in mitochondrial membrane potential without affecting astrocyte viability, however when these cells are further challenged with glutamate astrocyte viability and neuronal survival is compromised (Voloboueva et al., 2007). This is similar to the findings presented here which suggests that astrocytes fail to cope with an additional challenge when mitochondrial function is compromised by reduced signaling through IGF1R. Genis et al. (2014) have also previously reported that the IGF1 signaling pathway is important in preventing ROS production and supporting neuronal survival when challenged with oxidative stress in rodent astrocytes.

## Conclusion

Impairments in astrocytic IGF1 signaling affects their ability to protect neurons from oxidative stress, and is associated with dysregulation of energy-related metabolic pathways in astrocytes. Preserving or restoring IGF1 signaling in astrocytes to optimum levels might be an important translational goal to maintaining mitochondrial health, and hence neuronal support, during aging and stress, and this is relevant to a range of age-related neurodegenerative disorders.

## Author contributions

LER performed most of the experimental work and wrote the first draft of the paper. SBW conceived and supervised the study. CJG assisted with, and supervised, experiment design, planning, and collated the final version of the text and figures. IVV optimized culturing conditions for the LUHMES, performed the toxicity assay on the astrocytes and assessed neurite outgrowth in the presence of fibroblasts. LJ assisted with cell culture. PRH supervised the microarray experiments and data analysis. HM planned the metabolic assays and assisted with interpreting the findings and AS performed complex 1 assays in astrocytes. JES provided experiment supervision. EK assisted with generation of the GFP LUHMES. All of the authors discussed the interpretation of the findings and contributed to planning throughout the work. All of the authors commented and contributed to the paper.
